# Prevalence and Risk Factors for Hypertension among Myanmar Migrant Workers in Thailand

**DOI:** 10.3390/ijerph19063511

**Published:** 2022-03-16

**Authors:** Thin Nyein Nyein Aung, Yoshihisa Shirayama, Saiyud Moolphate, Thaworn Lorga, Warunyou Jamnongprasatporn, Motoyuki Yuasa, Myo Nyein Aung

**Affiliations:** 1 Department of Family Medicine, Faculty of Medicine, Chiang Mai University, Chiang Mai 50200, Thailand; drthinnyeinaung@gmail.com; 2 Department of Global Health Research, Graduate School of Medicine, Juntendo University, Tokyo 113-8421, Japan; shirayam@juntendo.ac.jp (Y.S.); moyuasa@juntendo.ac.jp (M.Y.); 3 Faculty of International Liberal Arts, Juntendo University, Tokyo 113-8421, Japan; 4 Department of Public Health, Faculty of Science and Technology, Chiang Mai Rajabhat University, Chiang Mai 50300, Thailand; saiyudmoolphate@gmail.com; 5 School of Nursing, Mae Fah Luang University, Chiang Rai 57100, Thailand; thaworn.lorga@gmail.com; 6 Provincial Health Office, Chiang Mai 50200, Thailand; warunyouj@gmail.com; 7 Advanced Research Institute for Health Sciences, Juntendo University, Tokyo 113-8421, Japan

**Keywords:** body mass index, hypertension, migrant workers, non-communicable diseases, Thailand

## Abstract

Background: Non-communicable diseases (NCDs) are showing an increasing trend worldwide, and the COVID-19 pandemic may interrupt or delay NCD care, the leading cause of mortality in Thailand, which is hosting 2–3 million migrant workers. The transition of epidemiological risk factors, limited access to health-promoting activities, and pandemic containment measures may adversely impact NCD risks. Therefore, hypertension and associated risk factors were determined among registered Myanmar migrant workers in Thailand. Methods: A cross-sectional survey with structured questionnaires was conducted in Thailand in 2017. Having hypertension was analyzed as a dependent variable, and the associated risk factors were explored by binary logistic regression analysis. Results: A total of 414 participants with a mean age of 29.45 ± 9.03 years were included, and 27.8 percent of the study participants were hypertensive, which was a rate higher than that in their host country (24.7%) and country of origin (26.4%). An older age, being male, current alcohol drinking, and being overweight and obese with reference to the body mass index (BMI) were significantly associated with hypertension. Conclusions: Our findings reaffirmed the idea that NCDs are important public health concerns, and a simple BMI measurement would be a valuable tool with which to determine hypertension risks. Targeted surveillance and an appropriate health policy are necessary for such a vulnerable population in Thailand.

## 1. Introduction

Non-communicable diseases (NCDs) are an increasing trend globally, and according to the World Health Organization (WHO), NCDs kill over 41 million people each year, equivalent to 71% of all the deaths worldwide. NCDs are chronic diseases that could affect the long and multifactorial origins of genetic, physiological, environmental, and behavioral factors. The WHO aims to reduce premature mortality from NCDs by one-third by 2030 [1]. The mortality from NCDs also ranks first in Thailand, and the mortality for all ages caused by prevalent NCDs is as follows: diabetes (4%), chronic pulmonary diseases (9%), cancer (17%), and cardiovascular diseases (CVDs) (29%), respectively—according to the WHO country profile for Thailand 2018 [1]. The highest proportion of mortality contributed by CVDs included stroke and ischemic heart diseases (IHDs), accounting for a quarter of all CVD deaths. The mortality from stroke has doubled, and that of IHD has increased by 50% in the past decade. Hypertension was attributed to two-thirds of stroke cases and half of IHDs in Thailand in 2017 [2]. Hypertension is a serious medical condition that significantly increases the risks of heart, brain, kidney, and other diseases. It is defined as systolic BP (SBP) ≥ 140 mmHg and/or diastolic BP (DBP) ≥ 90 mmHg, or reported treatment for hypertension [3]. The prevalence of hypertension is rising globally, and it is predicted to increase to 29.2% by 2025 [4]. Hypertension is one of the established modifiable risk factors for CVDs, and its prevalence is also increased in Thailand. According to the Thailand national health survey in 2014, one out of four Thai adults has hypertension, a disease named as a silent killer [5].

Thailand is currently hosting 2–3 million migrant workers from its neighboring countries such as Myanmar, Cambodia, and Laos [6]. Amongst them, Myanmar migrant workers comprised 80% of the total migrant population in Thailand. Moving to a country richer than their native country, they may adopt unhealthy lifestyle behaviors, which could affect exposures and vulnerability to NCD risk factors throughout their migration process, as highlighted by the International Organization for Migration [7]. Furthermore, upon return, some migrants arrive home less healthy than when they left, and the health care facilities there are limited. The burden of NCDs by migration is variable, and it depends on the migration status, country of settlement, and type of NCD [8]. In fact, modifiable behavioral risk factors for NCDs have been established, and much literature on preventing NCDs through lifestyle modifications, health education, and health-promoting activities may be limited in its accessibility for migrant workers. According to the Thailand Migration Report 2019, migrant workers are only screened for infectious diseases upon registration, and little is known about NCDs [9]. Even though NCDs are the number one cause of mortality in Thailand, screening for NCDs and long-term follow-up for chronic health conditions are still lacking among the migrant population. The affected individuals may be relatively young, and CVDs may not be a current problem. However, they may become a problem soon. The transition of epidemiological risk factors, limited accessibility to health-promoting activities due to the language barrier, and poor health education may impact their risks for NCDs. Moreover, their mobile nature and variable immigration status may determine the daily self-management of their diseases, continuing medical treatment, and follow-up visits to nearby health care facilities, subsequently affecting the complications of NCDs among this vulnerable population. The potentially higher burden of NCDs, by not being prevented or effectively controlled, could impact healthcare costs and the labor productivity of the host country. Therefore, it is important to determine migrant workers’ risky health behaviors to prevent prevalent NCDs such as hypertension. Our study aimed to determine the prevalence and associated risk factors for hypertension among migrant workers from Myanmar legally working in Thailand, since migration itself is a specific health challenge, and research focusing on the health and social security threats of migrant populations is consistently necessary to ensure a healthy global workforce.

## 2. Materials and Methods

### 2.1. Research Design

A cross-sectional survey using structured questionnaires was completed by face-to face interviews. Adult migrant workers (18 to 60 years old) who were willing to participate in the study by voluntarily giving written informed consent were recruited.

#### Population and Study Population

Sample size was calculated by using the Taro Yamane formulae, with reference to the total population of 81,299 Myanmar migrant workers in Chiang Mai province [10]. A sample size of 398 was calculated, and it was increased by 5% to compensate for incomplete data.

### 2.2. Research Instruments

Questionnaires were validated in the languages of targeted population, both in Thai and Myanmar versions. Following the WHO process of instrument translation and adaptation process, researchers investigated the readability and comprehension of the questionnaires by a pilot study including thirty migrant workers who voluntarily consented to participate. Pilot study participants were similar to the potential participants planned to be recruited in the future cross-sectional surveys in that they were (1) an immigrant person from Myanmar to Chiang Mai, Thailand, for labor; (2) ethnically either Myanmar or Shan; (3) either male or female gender; and (4) willing to participate in the research. The pilot study was conducted in March 2016 in Chiang Mai, Thailand [11]. The questionnaires were revised upon reviewing the results of pilot study to develop a final version. Validated questionnaires consisted of 3 parts to explore both modifiable and non-modifiable risk factors for hypertension: (1) socio-demographic characteristics, (2) health behaviors, and (3) measurements. Socio-demographic characteristics of the study participants included age, gender, history of chronic diseases such as diabetes, educational attainment (no formal education, primary school completed, and secondary school and above), marital status (single or married), types of job, and years of stay in Thailand. Regarding health behaviors, smoking and drinking alcohol, sleeping hours per day, and exercise habits were included. “Current smokers” were defined as those who smoked any tobacco products either on some days or every day. Former smokers or those who never smoked cigarettes were categorized as “current non-smokers”. Those who consumed any type of alcoholic drinks (spirit, beer, wine) regularly or irregularly during the previous year were categorized as “current alcohol drinkers”. Exercise activity was assessed: type of exercise such as walking, running, playing football, or playing badminton; duration of each exercise session; and number of exercise sessions per week were recorded. Measurements of height (m), body weight (kg), waist circumference (cm), hip circumference (cm), and blood pressure (mmHg) were completed.

### 2.3. Data Collection

The study participants were recruited with their written informed consent during the whole month of December 2017. The research team recruited the participants while they were waiting for the registration process at the provincial employment office, Chiang Mai. Data collection was completed using a stratified sampling technique and face-to-face interviews by the trained research assistants who were able to speak the languages of the study participants (Thai, Myanmar, and Shan languages).

A portable stadiometer was used to measure the standing height without shoes (m) and a weighing scale to measure the body weight without any jacket (kg). Body mass index (BMI) was determined by calculating weight (kg)/height (m^2^).

Using a standard measuring tape, measured to the nearest 0.1 cm, the midway between the lowest palpable rib and the anterior superior iliac crest was measured as waist circumference (cm), and the widest part of the buttock as hip circumference (cm). Additionally, then, waist/hip ratio was calculated to determine whether central obesity was present or not.

Digital sphygmomanometer was used to measure blood pressure. Following NICE guideline, measurement was repeated after taking rest for 5 min for those whose initial blood pressure measurement was over 140/90 mmHg to avoid white-coat hypertension [12].

### 2.4. Data Analysis

Data analysis was completed using SPSS version 24, and the final analysis included 414 participants. Socio-demographic variables were analyzed by descriptive analysis, and they included age (completed years), sex (male or female), ethnicity (Shan or Myanmar), marital status (single or married), and years of stay in Thailand. Educational status was categorized into three groups: no formal education, primary school completed, and completed secondary school and above. Type of jobs included no current employment; cleaning/household jobs; and construction, agriculture, or factory work.

According to WHO guidelines, BMI was categorized into three groups: Normal: BMI 18.5–24.9, Overweight: BMI 25–29.9, and Obese: BMI ≥ 30 [13]. 

Central obesity was determined by using WHO cutoff points for sex-specific waist/hip ratio. Participants with a waist/hip ratio ≥ 0.9 in males and ≥ 0.85 in females were regarded as having central obesity [14].

Exercise activity was grouped as “No exercise” for the study participants who never exercised or who had less than 150 min of moderate-intensity aerobic physical activity per week and “Exercise” for those who had at least 150 min of moderate-intensity aerobic physical activity or at least 75 min of high-intensity aerobic physical activity per week, following the WHO guidelines [15].

Blood pressure measurements were initially categorized into three groups: normo-tension: systolic blood pressure (SBP) < 120 mmHg and diastolic blood pressure (DBP) < 80 mmHg, pre-hypertension: SBP between 120–139 mmHg and/or DBP 81–89 mmHg, and hypertension: SBP ≥ 140 mmHg and/or DBP ≥ 90 mmHg, in accordance with hypertension screening in Thailand [16]. Thereafter, a dichotomous scale of “having hypertension” (SBP ≥ 140 mmHg and/or DBP ≥ 90 mmHg) or “no hypertension” (SBP < 140 mmHg or DBP < 90 mmHg) was recorded, and it was analyzed to be a dependent variable.

The basic statistical association between “having hypertension” and potential independent risk factors were initially evaluated by Chi-square tests. Variables with *p* values less than 0.7 were entered in the multivariable regression analysis to identify factors associated with “having hypertension”. Adjusted odds ratios (adjOR) with 95% confidence interval (95%CI) and *p* value ≤ 0.05 were considered to be significant associated factors.

## 3. Results

### 3.1. Characteristics of the Study Participants

The final analysis consisted of 414 participants, and their mean age was 29.45 ± 9.03 years. Male participants made up 55.8%, with females comprising 44.2%; 70.0% were married persons, and 49.0% did not have any formal school education, shown in Table 1.

Regarding associated NCDs, about 4.3% of study participants had never checked their diabetes status, and 1.0% had history of diabetes. Metabolic determinants of hypertension, such as the BMI and waist/hip ratio, along with behavioral determinants, such as current smoking, current alcohol drinking, and exercise habits, were assessed. About 15.2% of the participants exercised regularly; current smokers made up 26.3%; 40.8% had current alcohol-drinking habits; 68.1% did not have enough sleeping hours at night (less than 8 h); and more than 20% were overweight (16.4%)/obese (4.3%) (abnormal BMI), and 25% of participants had central obesity (raised sex-specific waist/hip ratio) (Table 1)

### 3.2. Prevalence of Hypertension and Associated Factors

**Figure 1:**
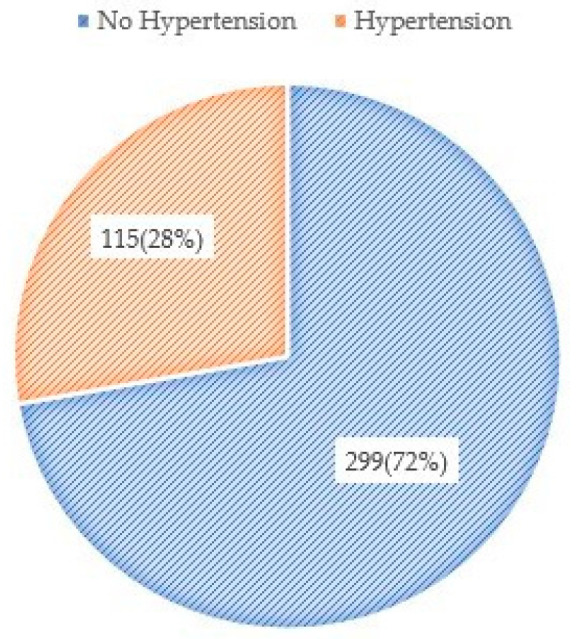
Prevalence of hypertension among Myanmar migrant workers in Chiang Mai, Thailand (2017).

About 27.8% of the study participants were hypertensive (Figure 1). The mean systolic blood pressure was 127.52 ± 18.24 mmHg, and the mean diastolic blood pressure was 83.13 ± 11.95 mmHg. When exploring the factors associated with hypertension, we noted that increased age was associated with an increased likelihood of having hypertension (AdjOR: 1.10, 95% CI: 1.07–1.13), and being male (AdjOR: 2.42, 95% CI: 1.12–5.24) compared to female, current alcohol drinking (AdjOR: 2.80, 95% CI: 1.41–5.57), and overweight (AdjOR: 5.88, 95% CI: 2.99–11.55) and obese (AdjOR: 6.10, 95% CI: 1.96–8.99), in terms of BMI, resulted in higher risk of hypertension (Table 2).

## 4. Discussion

We noted the important finding that 27.8% of the current study participants were hypertensive, a higher percentage than that of the host country (24.7%) and country of origin (26.4%) [1]. Hypertension was also more prevalent than in another study conducted among Shan migrant workers in Northern Thailand in 2011 (23.5%), comparable with the prevalence of hypertension among the Karen ethnic minority in Thailand (27.0%) and lower than that of South East Asian immigrants in the United States (29.1%) [17,18,19]. Age-related increases in blood pressure owing to vascular aging have been observed in almost every population, and we also noted that the increasing age of the study participants was associated with a higher prevalence of hypertension [20,21]. Distinct gender differences in the incidence and severity of hypertension were well-established, and hypertension was more common in men than women [22,23]. It is evident that blood pressure levels and hypertension increase with age in both sexes; however, men have higher blood pressure at a younger age than women [24,25]. Numerous previous observational epidemiological studies have supported that alcohol consumption can elevate blood pressure, and previous studies noted that males drank significantly more alcohol than females [26,27,28]. Being male per se is one of the non-modifiable CVD risk factors, and our finding of current alcohol drinking and its association with hypertension is an important but alarming issue in the goal of preventing hypertension by the modification of lifestyles among male migrant workers.

About 26.3% of study participants were current daily smokers, which was higher than Thailand’s 2021 national figure of 17.0% and that of Myanmar of 15.0% [29]. The effects of tobacco smoking on hypertension are complex, and it is evident that smoking raises blood pressure over time. Moreover, both smoking and hypertension act as independent risk factors for cardiovascular diseases [30,31]. Even though we did not find any significant association of current smoking status with hypertension, the higher prevalence of current daily smokers in this study should be considered for the prevention of NCDs and subsequent burdens. Furthermore, less than a quarter of participants had regular exercise habits (15.2%), and the proportion of physical inactivity in this study population (84.8%) was also higher than that of the host (25.0%) and native country (10.0%) [1]. One-third of study participants could have adequate sleeping hours (>8 h per night). However, hypertension was not significantly affected by “no exercise” and lack of sleep in this study.

The association of obesity with hypertension was explored both in terms of central obesity by the sex-specific waist/hip ratio and overweight/obesity by the BMI. We did not find any significant association of central obesity with hypertension whereas the overweight and obese study participants (in terms of the BMI) had significantly higher association with hypertension than those with a normal BMI. The risk of hypertension among overweight participants was as high as among obese ones (about six times higher than their normal BMI counterparts). Pre-existing literature reported that excessive body weight (high BMI) is an important determinant of hypertension, and obese people had a higher risk of developing hypertension when compared to people with normal weight. This obesity-associated hypertension may be due to abnormal kidney function with a subsequent increase in blood pressure [32,33,34].

Our findings highlighted the prevalence of hypertension and its associated behavioral risk factors among young, working migrants, to prevent NCDs, which would be valuable in maintaining a healthy workforce and increasing productivity in a host country. Moreover, currently, the burden of the COVID-19 pandemic may affect NCDs. As of November 2021, over 260 million confirmed COVID-19 cases and nearly 5.2 million deaths have been reported globally. Persons with chronic diseases, such as NCDs and other diseases, have a greater risk of severe COVID-19, probably leading to higher mortality or prolonged hospitalization. Additionally, the postponement or interruption of NCD care and pandemic containment measures such as lockdown and social distancing may increase unhealthy lifestyle behaviors, such as poor diets, alcohol, and physical inactivity, which may constitute increased risks for NCDs and subsequent negative impacts on morbidity and mortality [35].

### Limitation and Strength of This Study

The generalizability of our findings to represent all Myanmar migrant workers in Thailand is affected by the fact that most of the study participants are ethnic Shan due to the geographical proximity between Chiang Mai, Thailand, and the Shan state of Myanmar. Another limitation of the present study is that information about poor diet, especially salt intake, is missing. Assessment of renal function to exclude an important disease underlying hypertension was not included in this cross-sectional survey, adding one more limitation to this study. The family history of hypertension could not be explored, as participants could neither remember the history of their family nor realize the importance of such family history. The prevalence of hypertension, as determined by the objective assessment of resting blood pressure in a crowded data-collection site and repeating BP measurements a second time only for those whose initial BP was over 140/90 mmHg, may limit the main outcome of this study. However, reaching a vulnerable population with well-validated questionnaires and well-trained research assistants who were able to speak three languages (Thai, Shan, and Myanmar) of the targeted study population strengthened the valuable findings of our study.

## 5. Conclusions

Despite the limitations, our study reaffirmed that NCDs are important public health concerns among the migrant population. Moreover, the measurement of BMI to determine hypertension risks would be a valuable, easy, and simple assessment tool. Our findings also provided insights into the epidemiological patterns of risk behaviors of hypertension, which could impact the negative consequences of NCDs and the global workforce. Even though they share the same NCDs risk factors with people of the host country and those of the native country, they may have more potential risks due to lack or inaccessibility of health care services in Thailand. This may put them at risk of developing hypertension and an increased burden of NCDs while in the host country and once they return home. Targeted surveillance and urgent health policy are recommended for this migrant population in Thailand by strengthening partnerships in cross-border and international global health.

## Figures and Tables

**Table 1:** Characteristics of the study participants (*n* = 414).

	Hypertension		
Variables	Yes (*n*%)	No (*n*%)	Total (*n*%)
Age			29.45 ± 9.03 years
Sex			
Male	84 (73.0)	147 (49.2)	231 (55.8)
Female	31 (27.0)	152 (50.8)	183 (44.2)
Marital status			
Single	25 (21.7)	99 (33.1)	124 (30.0)
Married	90 (78.3)	200 (66.9)	290 (70.0)
Education			
No formal education	61 (53.0)	142 (47.5)	203 (49.0)
Primary school completed	32 (19.1)	81 (27.1)	113 (27.3)
Secondary school and above	22 (19.1)	76 (25.4)	98 (23.7)
Years of stay in Thailand			6.36 ± 5.70 years
Types of job			
Cleaning/household works	29 (25.2)	97 (32.4)	126 (30.4)
Construction	43 (37.4)	82 (27.4)	125 (30.2)
Agriculture	29 (25.2)	80 (26.8)	109 (26.3)
Factory	13 (11.3)	26 (8.7)	39 (9.4)
Currently unemployed	1 (0.9)	14 (4.7)	15 (3.6)
Diabetes			
Yes	2 (1.7)	2 (0.7)	4 (1.0)
No	109 (94.8)	283 (94.6)	392 (94.7)
Never checked	4 (3.5)	14 (4.7)	18 (4.3)
Sleeping hours per night			
<8 h	83 (72.2)	199 (66.6)	282 (68.1)
>8 h	32 (27.8)	100 (33.4)	132 (31.9)
Exercise			
No	96 (83.5)	255 (85.3)	351 (84.8)
Yes	19 (16.5)	44 (14.7)	63 (15.2)
Current smoking			
No	69 (60.0)	36 (78.9)	305 (73.7)
Yes	46 (40.0)	63 (21.1)	109 (26.3)
Current alcohol drinking			
No	46 (40.0)	199 (66.6)	245 (59.2)
Yes	69 (60.0)	100 (33.4)	169 (40.8)
Central obesity			
No	79 (69.3)	231 (77.3)	310 (75.1)
Yes	35 (30.7)	68 (22.7)	103 (24.9)
Body Mass Index (BMI)			
Normal 18.5–24.9	68 (59.1)	260 (87.0)	328 (79.2)
Overweight 25–29.9	37 (32.2)	31 (10.4)	68 (16.4)
Obese ≥ 30	10 (8.7)	8 (2.7)	18 (4.3)

**Table 2:** Factors associated with hypertension among Myanmar migrant workers in Thailand.

	Hypertension			
		95% Confidence Interval		
	*n* (%)	Adjusted OR	Lower	Upper
Age (years)		1.10 **	1.07	1.13
Sex				
Female	31 (16.9)	Referent		
Male	84 (36.4)	2.42 *	1.12	5.24
Current alcohol drinking				
No	46 (18.8)	Referent		
Yes	69 (40.8)	2.80 *	1.41	5.57
Current smoking				
No	69 (22.6)	Referent		
Yes	46 (42.2)	1.21	0.64	2.29
Exercise				
Yes	19 (30.2)	Referent		
No	96 (27.4)	1.26	0.62	2.56
Central obesity				
No	79 (25.5)	Referent		
Yes	35 (34.0)	1.18	0.61	2.29
Body Mass Index				
Normal 18.5–24.9	68 (20.7)	Referent		
Overweight 25–29.9	37 (54.4)	5.88 **	2.99	11.55
Obese ≥ 30	10 (55.6)	6.10 **	1.96	18.99

* *p* value ≤ 0.05; ** *p* value ≤ 0.01.

## Data Availability

The data will be available from the corresponding author upon a reasonable request.
